# Novel application of orthopedic suture tape for sternal closure after upper hemisternotomy

**DOI:** 10.1093/jscr/rjaf343

**Published:** 2025-05-23

**Authors:** Vishal N Shah, Joshua R Chen, Konstadinos A Plestis

**Affiliations:** Division of Cardiothoracic Surgery, Thomas Jefferson University Hospital, 1025 Walnut Street, College Bldg, Ste 607, Philadelphia, PA, United States; Division of Cardiothoracic Surgery, Thomas Jefferson University Hospital, 1025 Walnut Street, College Bldg, Ste 607, Philadelphia, PA, United States; Division of Cardiothoracic Surgery, Thomas Jefferson University Hospital, 1025 Walnut Street, College Bldg, Ste 607, Philadelphia, PA, United States

**Keywords:** upper hemisternotomy, FiberTape closure, suture tape, stainless steel wires, aortic valve replacement

## Abstract

The FiberTape (FT) System (Arthrex, Naples, FL, USA), initially developed for orthopedic surgery, is a new, nonmetallic, suture-based alternative to standard stainless-steel wires for sternal reapproximation. Publications on FT use after cardiothoracic surgery are limited. The FT System has not been used in upper hemisternotomy (UHS) closure. Consequently, we report the use of the FT System for sternal closure in a 70-year-old man after UHS aortic valve replacement and discuss several advantages of its application.

## Introduction

The FiberTape (FT) System (Arthrex, Naples, FL, USA) is a new cerclage tape originally developed for long bone fracture fixation and ligament reconstruction. The FT System has recently expanded into cardiothoracic surgery as an entirely nonmetallic, suture-based alternative to standard stainless-steel wires (SSW), providing strong, homogenous, and reproducible compression for sternal reapproximation and lowering the risk of bone cut-through [1, 2]. Publications on FT use after cardiothoracic surgery are sparse. Specifically, FT has been used in bilateral transverse thoracosternotomy and full sternotomy patients [1, 2]. It has not been reported in upper hemisternotomy (UHS) closure. Thus, we document the application of the FT System for sternal closure after UHS aortic valve replacement (AVR) to accentuate several advantages of its application. Patient consent for publication was obtained.

## Case report

**Figure 1:**
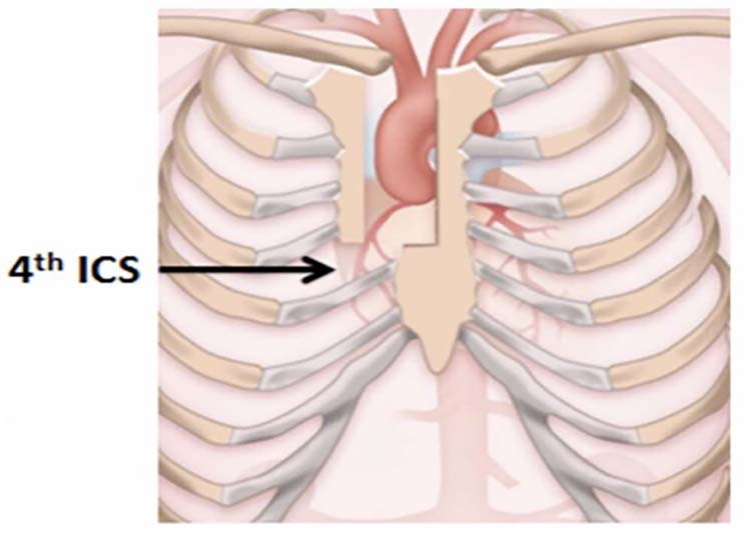
Upper hemisternotomy incision.

**Figure 2:**
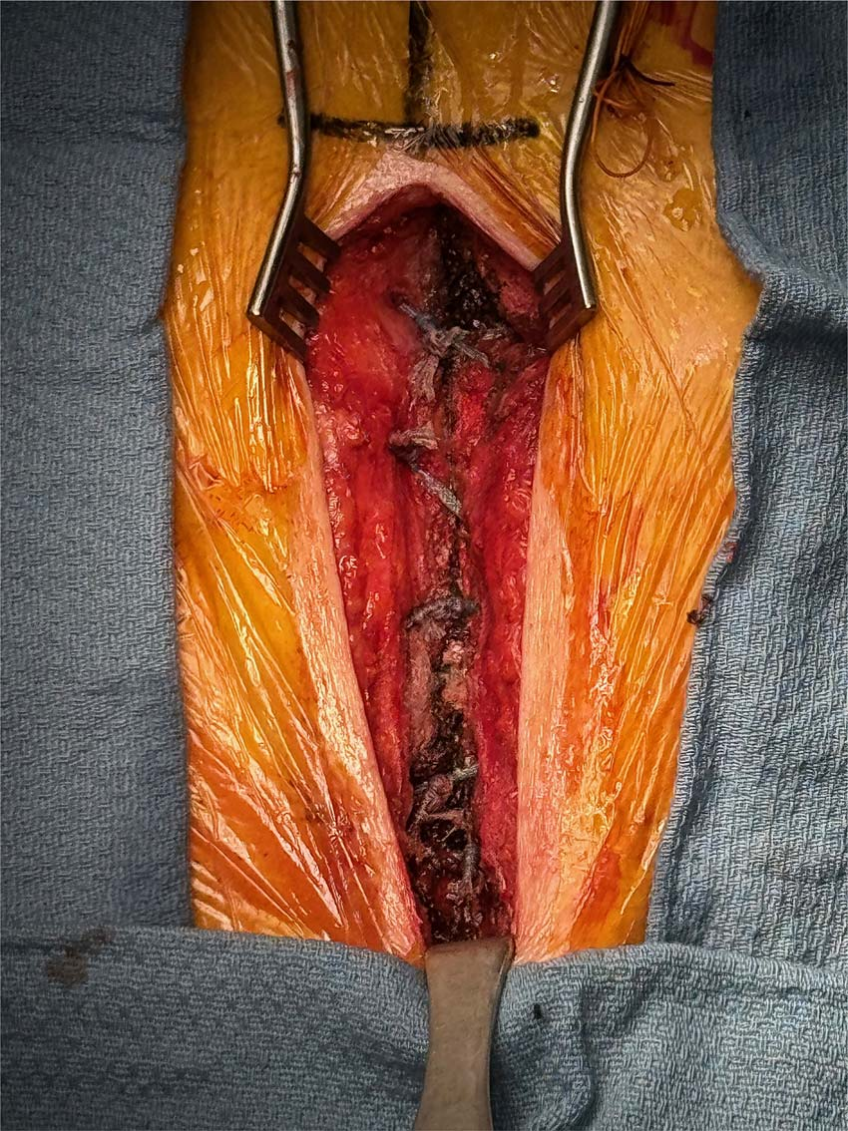
Upper hemisternotomy closure with FiberTape.

A 70-year-old man with severe symptomatic aortic stenosis with a mean pressure gradient >40 mmHg and an aortic valve area <1 cm^2^ presented for AVR. Our UHS approach to AVR has been previously published in detail [3–5]. A J-shaped UHS was performed 2 cm inferior to the sternal notch to the midpoint of the 4th intercostal space and exiting the right lateral 4th intercostal space (Fig. 1). Ascending aortic and femoral venous cannulation were performed using the Seldinger technique under transesophageal echocardiographic guidance. A single dose of antegrade del Nido cardioplegia was administered after aortic cross-clamping. Subsequently, biological AVR was performed in a standard fashion. At closure, the manubrium and sternal body were loosely reapproximated with 2 and 3 interrupted tapes, respectively. Each of the tape ends were then brought through a performed loop and hand tightened down closing the sternum loosely. A tensioner was placed on each tape and incrementally tensioned to 60–80 Newtons. Once satisfactory tension of 60–80 Newtons across all tapes was achieved, the tape ends were tied together for 3 alternating knots and suture tails were then cut. The completed intraoperative closure is shown in Fig. 2 and Video 1. The muscle and subcutaneous tissues were closed in layers with absorbable suture, and the skin was stapled. Per routine, a negative pressure dressing (Prevena, KCI/3M, San Antonio, TX, USA) was placed over the incision. The patient was discharged on postoperative day 6 and has a well-healed sternal incision 12 months after AVR.

## Discussion

The FT System is constructed from a 2 mm wide nonabsorbable, multistrand, long-chain, ultra-high-molecular-weight polyethylene core with a braided jacket of polyester, delivering biomechanical superiority in published in vitro and in vivo studies [6, 7]. The FT System exhibits higher mechanical strength and can resist almost double the force compared to SSW in addition to demonstrating less stretching and a high modulus of resilience [8, 9]. In lung transplant recipients undergoing bilateral transverse thoracosternotomy, Coster et al. observed no short-term sternal complications in 22 patients closed with FT versus 8 (28.6%) sternal complications in 28 patients closed with SSW (*P* = .006) [1]. Similarly, in 45 patients undergoing full sternotomy closure with FT after cardiac surgery, DiGiorgi noted no early failures or sternal wound infections and also found FT closure was faster than SSW closure [2].

The UHS incision is popular. We employ UHS for all elective replacements of the aortic valve and root, ascending aorta and hemiarch and have published extensively on it [3–5]. Although the optimal UHS closure technique remains uncertain, SSW is routinely used. To date, FT cerclage has not been reported after UHS. We believe that due to space and mobility constraints from a smaller incision especially in obese patients, UHS closure can be more cumbersome than full sternotomy closure when using SSW. Hence, FT, being flexible and braided, can be easier to work with compared to the stiffer SSW, provides greater elasticity without losing stability, and less likely to cut through bone due to its fabric reinforcement [1, 2, 6–9]. An assortment of advantages to FT also includes reducing soft tissue irritation, avoiding the risks of metal-related allergies, hardware prominence and failure with subsequent chest wall pain and injury from sharp SSW ends, allowing to be easily cut with Mayo scissors or a scalpel for quick reentry, applying a measurable and consistent amount of tension to the sternum by using a tensioner, and ensuring excellent reproducibility and repeatability by removing the human factor of forced twisting and tightening of SSW [1, 2, 6–9]. In light of greater scrutiny over sternal stability and wound healing in The Enhanced Recovery After Cardiac Surgery guidelines, SSW alternatives have been advocated to promote optimal fixation and stabilization of healing bone [10]. Consequently, we speculate FT use particularly with minimally/less invasive cardiac surgery may work together to hasten a patient’s recovery. To conclude, FT can be safe and efficient for UHS closure.

## Supplementary Material

Video_1_rjaf343
